# Acute Surgical Conditions of the Abdomen Often Mistaken for Appendicitis

**Published:** 1911-08

**Authors:** J. L. Campbell

**Affiliations:** Professor Surgical Anatomy and Clinical Surgery, Atlanta School of Medicine, Visiting Surgeon, Wesley Memorial Hospital and Tabernacle Infirmary


### ACUTE SURGICAL CONDITION OF THE ABDOMEN OFTEN MISTAKEN FOR APPENDICITIS.

By J. L. Campbell, M. D.

*Professor Surgical Anatomy and Clinical Surgery, Atlanta School of Medicine, Visiting Surgeon, Wesley Memorial Hospital and Tabernacle Infirmary.*

The Appendix is so frequently the cause of acute abdominal pain and tenderness that we are loath to consider any other cause, but when the abdomen is opened and an appendix normal in appearance is found it becomes our duty to enlarge the incision and make a thorough search of the abdominal cavity for some other lesion.

There are perhaps three or four conditions that may deceive us that, like acute appendicitis, require immediate surgical interference. It is often impossible to distinguish the one from the other unless we are very careful at the time of operation.

If I may be permitted to suggest the order of frequency with which such conditions occur, I shall make the following classification: 1st, intestinal obstruction; 2nd, intestinal perforation; 3rd, Female pelvic lesions, (a) extra-uterine pregnancy, (b) acute suppuration salpingitis or (c) an ovarian cyst, large or small, twisted on its pedicle; and 4th, rupture of the gall bladder.

If I were to discuss each of these as it deserves, it would prolong this paper far beyond the time allotted me. I will therefore mention some of the points that appeal to me most and report two cases that have occurred in my practice, and mention one or two others that have been brought to my attention.

1st. Intestinal obstruction, like acute appendicits, is accompanied by pain, rigidity of the abdominal muscles, shock nausea, etc, and, when occuring in a person hitherto healthy, will naturally be unrecognized.

The etiology of this condition will probably be found in the following order: (a) adhesions to a Meckel's diverticulum; (b) volvulus; (c) intussusception; (d) an unrecognized hernia—diaphragmatic, obturator, or into some one of the posterior fossa of the peritoneal cavity; and (e) strangulation from adhesions following trauma or peritonitis.

Volvulus is perhaps the easiest of the above to overlook and is perhaps the cause of many deaths following operations for acute appendicitis. This is at present quite vivid in my mind, as I found a volvulus some distance from an acutely inflamed appendix in a case recently referred to me by Dr. Niles. I was about to close up the incision when I noticed a collapsed portion of the intestine and following it up found a volvulus with the gut involved quite dark.

2nd. Perforations may occur: (a) as result of an ulcer of the duodenum; (b) acute diverticulitis; or (c) during the latter part of or convalescence from typhoid fever—here it would hardly be mistaken for any other disease.

Perforations, like obstructions, give practically the same symptoms as those of the first stage of acute appendicitis and will also be accompanied by the same blood changes.

I want to again urge the importance of making further investigation where there is an appendix of normal appearance and yet the patient has had pain, shock, and other indications of severe abdominal lesions. Had this rule been adhered to in the past, perhaps more than one life would have been saved.

Much has been said recently in reference to perforating ulcer of the duodenum and the frequency with which it may be mistaken for acute appendicitis, yet within the past year a boy about ten years old was operated on at one of our hospitals for what was supposed to be an acute appendicits—the post-mortem showed a perforating ulcer of the duodenum.

Within the past few months much has been written about diverticulitis and several interesting cases have been reported. Within the past decade certain pouchings of the intestines, varying in size from those that would scarcely admit the point of a fine probe, to the size of a small pencil, have been noticed by surgeons and pathologists. These pouches are usually situated at the mesenteric attachment and are most frequently found at or near the sigmoid, though they may exist at any portion of the intestinal canal. These diverticulae may be congenital or due to pressure from within the canal; they may have all the coats of the intestines or only the mucous and peritoneal, from which it would seem that they are due to pressure. They are more frequent in the aged, though several cases have been reported in the young. Like the appendix, they are subject to inflammations and ruptures, and of this latter condition I will report a case on which I operated about two months ago.

H. D., male, age nineteen, was admitted to the Wesley Memorial Hospital with a diagnosis of acute appendicitis of about thirty hours' duration. I made a hurried examination and found the abdominal wall rigid, pain and tenderness in the right lower quadrant. I did not go into the history at the time, for he had been seen by three of our best doctors and all had recommended an operation. The pain was excruciating and there was every symptom of a grave abdominal lesion.

I made a rather long incision at McBurney's point. When the cavity was opened a quantity of milky pus was evacuated. The appendix showed no sign of inflammation, although bathed in the pus. It was removed and a further examination made to see if there were any adhesions, as a portion of the small intestine presenting was very dark. It was found strangulated in two places by recently formed bands of adhesions, which were broken up. The pelvis contained about a tea cup full of the thin milky pus, which was wiped out. In retracting the intestines for our first exploration, the House Surgeon, Dr. Wood, who was assisting me said that he felt something harder than usual about two inches above the cecum on the inner side of the ascending colon. On examination I found that it was a pouched out mass at the mesenteric attachment which had ruptured. There was considerable thickening of the intestinal wall around it. The contents of the colon were escaping. The cavity was wiped as free of this matter as possible and abundant drainage was placed around the rupture and walled off as best we could with rubber dam. Two cigarette drains were carried into the pelvis from the lower angle of the incision. The wound was closed only sufficiently to cover the intestines. The patient placed in the Fowler position and proctocysis was given for forty-eight hours. The drains were removed in seventy-two hours. He made an uneventful recovery, and with the exception of an hernia at the site of the incision is well at this time. The only thing in the past history that would throw any light on the case, if that did, was an attack of typhoid fever about two years ago. There had been some slight pain for a day or two before the sudden onset of the present attack.

3rd. Sudden pain, shock, and rigidity of the abdominal muscles are perhaps more often due to the twisting of a pedunculated pelvic tumor than to suppurative salpingitis, and, if there is the slightest doubt about the appendix, the pelvis should be examined. In this class of cases I have had no personal experience, but I have known of one or two cases occurring in Atlanta. It is not likely that an extra-uterine pregnancy will be overlooked if any case is taken.

Sudden attacks of pain from suppuration salpingitis are rare, but it may occur where least expected. Miss L. S., age about twenty-three, was feeling as usual and spent the evening with the family. Shortly after retiring she was attacked by sudden and violent pain in the lower abdomen. Simple remedies failed, and I was called early in the morning. I found the abdominal wall rigid and so tender that the weight of the hand was borne with difficulty. Morphine gave only temporary relief. I called my friend Dr. Hunter P. Cooper, and he agreed with me that an operation should be made as soon as possible, as he believed the appendix had ruptured but cautioned me to examine the right tube.

She was carried as soon as possible to the Tabernacle Infirmary, where seventeen hours after the beginning of the attack I opened the abdomen, making an incision at McBurney's point. The intestines were bathed in a cream colored pus. The appendix was slightly darker than normal, but not perceptibly involved; it was removed. As we were making some manipulations the fimbriated extremity of the Fallopian tube appeared at the lower angle of the wound and pus was noticed escaping from it. On pressure quite a quantity escaped. The tube was pulled up, and it and the ovary removed. The left tube was also pulled up and found to contain pus. The incision at McBurney's point was closed and one made in the median line through which the left tube was removed, and the pelvis wiped out. There were no adhesions and no lymph on the peretoneum. Drainage was introduced through the vagina and the lower end of the median incision and one wick carried over to the appendix stump.

She did well, and her temperature was normal in about five days and remained so for four or five more, when it began to rise gradually. There was no indication of infection of any of the incisions and no pain or fullness to be found by a vaginal examination. The temperature continued to rise and followed a typical typhoid fever course for four weeks, when convalescence was established and she made a slow recovery.

4th. Rupture of the gall bladder will be perhaps the most easily recognized of any condition enumerated above because of the bile stain if the cystic duct has not been obliterated.

In conclusion, I wish to again emphasize the importance of exploring the abdominal cavity if we are not absolutely certain that the appendix is the only structure involved. In order to make sure of this we are compelled to make a somewhat longer incision than is advocated by some, and I believe that we can accomplish best what we wish by making the incision along the outer border of the rectus muscle.

